# Tannic Acid-Modified Sodium Caseinate Pickering Emulsion Coatings: Characterization, Enhanced Mechanical/Antibacterial Properties, and Application in Cherry Tomato Preservation

**DOI:** 10.3390/foods14183190

**Published:** 2025-09-12

**Authors:** Qiyuan Feng, Hesheng Wang, Xinyu Yang, Linna Wang, Tian Li, Limin Guo, Silong Jia, Yaqian Yang, Youwei Yu, Shaoying Zhang

**Affiliations:** College of Food Science, Shanxi Normal University, Taiyuan 030000, China; 15713577244@163.com (Q.F.); wanghesheng2025@163.com (H.W.); 223420014@sxnu.edu.cn (X.Y.); 223420011@sxnu.edu.cn (L.W.); 223420028@sxnu.edu.cn (T.L.); 223420013@sxnu.edu.cn (L.G.); 223420006@sxnu.edu.cn (S.J.); 224420009@sxnu.edu.cn (Y.Y.)

**Keywords:** coatings, carboxymethyl tara gum, Pickering emulsion, cherry tomato, fresh-keeping

## Abstract

This study developed a tannic acid-modified sodium caseinate (SC-TA) stabilized Pickering emulsion containing bergamot essential oil (BEO) and carboxymethyl tara gum for cherry tomato preservation. Fourier transform infrared (FTIR) spectroscopy and circular dichroism (CD) analysis confirmed successful SC-TA conjugation and improved emulsion stability. The emulsion significantly lowered the water vapor permeability (WVP) of the film, enhanced its tensile strength and elongation, and exhibited antimicrobial activity against *Escherichia coli* and *Staphylococcus aureus* (the inhibition zones of the coating with Pickering emulsion were 10.67 mm larger and 6.67 mm larger than those without Pickering emulsion, respectively, against *Escherichia coli* and *Staphylococcus aureus*), as well as antioxidant capabilities (the coating with Pickering emulsion showed a 128.6% increase in DPPH scavenging rate and a 341.8% increase in ABTS scavenging rate compared to the coating without Pickering emulsion). Applied to cherry tomatoes, it effectively reduced quality deterioration by minimizing weight/firmness loss, preserving nutrients (vitamin C, lycopene), and decreasing oxidative damage. These comprehensive effects confirm that the BEO-stabilized Pickering emulsion coating represents a promising technology for postharvest management, capable of extending fruit shelf life while preserving nutritional quality.

## 1. Introduction

Cherry tomatoes are nutritionally dense, containing significant amounts of bioactive compounds including carotenoids, vitamin C, lycopene, and polyphenolic compounds. These phytochemicals have been shown to exhibit potential chemopreventive effects against certain malignancies and cardiovascular disorders [1]. However, cherry tomatoes are very easy to soften and deteriorate due to respiratory activity and pathogen invasion during the ripening, resulting in a large amount of loss after harvest. Among these, black mold caused by *Alternaria* is the main postharvest disease of cherry tomato fruits [2]. Multiple preservation approaches have been investigated for maintaining postharvest produce quality, including high-pressure processing (HPP), low-temperature storage, modified atmosphere packaging, chemical preservatives, and antimicrobial active packaging systems. While these methods demonstrate efficacy in reducing the deterioration of cherry tomatoes, their practical application is often limited by high implementation costs, scalability challenges, and potential safety concerns regarding human consumption [3].

Edible coatings represent an innovative approach to food preservation, utilizing naturally derived biopolymers from animal, plant, or microbial sources. These eco-friendly alternatives offer significant advantages for extending the shelf life of perishable produce while addressing environmental concerns. Edible coatings are typically formulated using three major classes of biopolymers: polysaccharides, proteins, and lipids. Common polysaccharide-based materials include chitosan, pectin, starch derivatives, alginates, amylopectin, carrageenan, and modified celluloses. These biomaterials offer diverse functional properties for food packaging applications [4]. Tara gum (TG), derived from the endosperm of *Caesalpinia spinosa* seeds indigenous to South America, represents a cost-effective galactomannan polysaccharide with broad agricultural cultivation. Structurally, it consists of a β-(1 → 4)-linked d-mannopyranose backbone with α-(1 → 6)-d-galactopyranose side groups. This natural hydrocolloid is predominantly utilized in the food industry as a viscosity modifier and colloidal stabilizer due to its excellent water-binding capacity and rheological properties [5]. However, the aqueous solution of TG exhibits high viscosity [6], which is not suitable for coating. Nevertheless, Barreto Santos et al. [7] demonstrated that depolymerization of the main chain of TG during carboxymethylation lowers the average molecular weight; this reduction effectively attenuates solution viscosity, resolving the excessive consistency that otherwise precludes the formation of homogeneous edible coatings.

Carboxymethyl tara gum lacks inherent antimicrobial and antioxidant, so to work with some antimicrobial and antioxidant substances to achieve effective preservation is needed. Essential oils have garnered significant scientific interest due to their demonstrated bioactivities, including antimicrobial efficacy, radical scavenging capacity, and therapeutic potential in pharmacological applications [8]. Bergamot essential oil (BEO), obtained through steam distillation of Citrus bergamia fruit rind, exhibits multiple bioactive properties, including antimicrobial, antitumor, neuroprotective, and free radical-scavenging capacities, making it valuable for diverse industrial applications. This essential oil is used in a wide range of applications in food processing, medicinal formulations, fragrance development, consumer products, and personal care formulations. Apart from its chemical composition, the unique aromatic flavor of BEO also has the potential to mitigate the flavor degradation during the storage of fresh fruits and vegetables, and it has an aroma that can be compatible with the odor of tomatoes. Research indicates that (+)-limonene and (−)-linalool serve as the primary bioactive constituents responsible for BEO’s characteristic fragrance and physiological effects, existing predominantly in their respective enantiomeric forms [9]. Chen Xing [10] and Susana Ribes [11] et al. found that BEO had inhibitory effects on *Staphylococcus aureus*, *Escherichia coli*, *Saccharomyces cerevisiae*, and *Aspergillus Niger*. However, essential oils are almost insoluble in water-based systems and can only be effectively incorporated into biopolymer-based coatings through specialized techniques. Pickering emulsions represent a class of stabilized colloidal systems where biopolymeric particles irreversibly adsorb at the oil-water interface. In these systems, bioactive essential oils are encapsulated within the emulsion droplets, enabling controlled release while simultaneously providing antimicrobial and antioxidant functionalities. This unique stabilization mechanism differs fundamentally from conventional surfactant-stabilized emulsions, offering enhanced stability against coalescence [12].

Through a series of structural and chemical modifications, proteins can assemble into a dense interfacial layer around the dispersed droplets, thereby stabilizing the Pickering emulsion as a solid particle [13]. Sodium caseinate (SC) was selected as the primary stabilizer for this investigation due to its cost-effectiveness and widespread availability in food applications. This milk-derived protein exhibits rapid adsorption at hydrophobic interfaces, forming an interfacial layer that effectively stabilizes oil-in-water emulsions through combined electrostatic repulsion and steric hindrance mechanisms. In recent years, SC particles were prepared using complexation with phenolic compounds, glycosylation, and antisolvent methods with new functional properties. Therefore, the production of modified SC particles with new capabilities, including the ability to stabilize the emulsion with a higher oil phase percentage, is very valuable [14]. Tannic acid (TA) is a kind of plant-derived polyphenol with multiple hydroxyl groups (around 25 hydroxyl groups) in its structure, providing abundant interaction sites with biomacromolecules (such as protein, polysaccharides and mixtures). TA can form complexes with proteins through its hydroxyl groups and phenolic rings, which improves the interfacial functionalities of proteins [15]. Previous research by Nourabi et al. [14] has demonstrated the efficacy of SC-tannic acid (TA) complexes in Pickering emulsion stabilization, providing a scientific basis for the current study’s approach. To the best of our knowledge, few studies have employed SC–TA complexes as Pickering stabilizers for the preservation of fresh fruits. Building upon these findings, our current work employs SC-TA nano complexes as particulate stabilizers for Pickering emulsion formulation. And apply it to preserve fresh fruits.

To the best of our knowledge, this is the first report in which carboxymethyl tara gum is employed as the film-forming biopolymer for the fabrication of an edible coating. This is also the first time that the stable Pickering emulsion of SC-TA nanocomposite has been applied to fruit preservation. The carboxymethyl tara gum matrix was integrated with a SC–TA complex-stabilized Pickering emulsion encapsulating BEO. The resulting composite coating may utilize the continuous release effect of BEO to inhibit the physiological metabolic process of cherry tomatoes after harvest and prevent microbial spoilage. The ultimate goal of this study is to provide a theoretical basis for the application of carboxymethyl tara gum in fruit coating and explore the potential of bergamot essential oil in fruit preservation. This research offers new possibilities for the preservation application of postharvest fruits.

## 2. Materials and Methods

### 2.1. Materials

Tara gum was purchased from Zhejiang Yinuo Biotechnology Sample store (Quzhou, China). Sodium caseinate was purchased from Shanghai Maclin Biochemical Technology Co., Ltd. (Shanghai, China). Tannic acid was purchased from Shanghai Yuanye Bio-Technology Co., Ltd. (Shanghai China). Bergamot essential oil was purchased from doTERRA (Shanghai) Trading Co., Ltd. (Shanghai, China). Sodium hydroxide (NaOH) and glycerol was supplied by Tianjin Fangda Tongzheng Chemical Co., Ltd. (Tianjin, China). Monochloroacetic acid (MCA, analytical grade) and 2,2′-casino-bis (3-ethylbenzothiazoline-6-sulfonic acid) was obtained from Shanghai Aladdin Biochemical Technology Co., Ltd. (Shanghai, China). 1,1-diphenyl-2-picrylhydrazyl (DPPH) were sourced from Tokyo Chemical Industry Co., Ltd. (Tokyo, Japan). *Alternaria alternata* (BNCC 115062), *Escherichia coli* (ATCC 11229), and *Staphylococcus aureus* (CMCC 26003) were purchased from Beijing Nabio Biotechnology Co., Ltd. (Beijing, China). Lycopene (96%) was purchased from Liaoning Ku Kou Biotechnology Co., Ltd. (Shenyang, China). Cherry tomatoes (Millennium variety) purchased from Jinzhong Huilong Farmers market (Xi’an, China).

### 2.2. Preparation and Characterization of Nanocomposites and Pickering Emulsions

#### 2.2.1. Preparation of SC-TA Nanocomposites

**Scheme 1:**
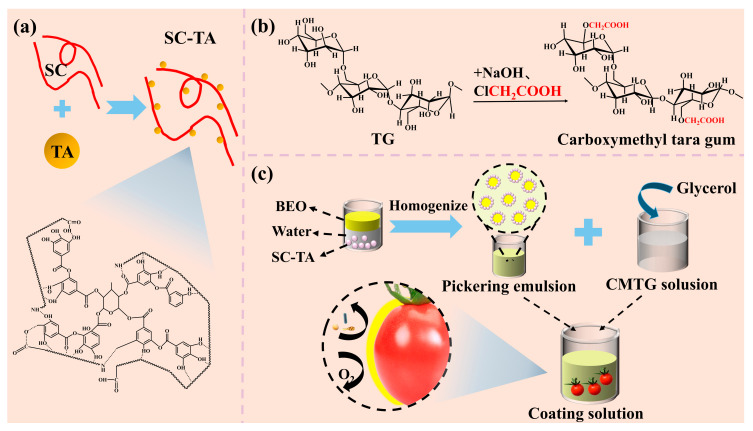
Material Preparation and Preservation Process Flow Diagram. (**a**) The schematic representation of the SC–TA nanocomposite structure. (**b**) The chemical structure of TG before and after its carboxymethylation. (**c**) The simple preparation flowchart of the coating solution.

The SC-TA nanocomposites were prepared according to Zhan et al. [16]. Precise amounts of SC and TA were weighed to achieve liquid-to-solid ratios of 1 mL g^−1^ (SC) and 10 mL g^−1^ (TA), respectively, while maintaining a mass ratio of SC:TA = 1:0.1. This ratio was optimized via preliminary experiments, at which the resulting Pickering emulsion exhibited the highest storage stability. Both components were subsequently dispersed in phosphate buffer (pH 6.0, 0.01 M) at 25 °C under continuous stirring until complete dispersion was achieved. The pH of the buffer solution was adjusted using HCl (0.01 N) or NaOH (0.01 N). Then the mixture was stored overnight at 4 °C. Scheme 1a presents a schematic representation of the SC–TA nanocomposite structure.

#### 2.2.2. Preparation of Tannin-Sodium Caseinate Stabilized BEO Pickering Emulsion (PE)

The SC-TA nanocomplex suspension, stored overnight, was centrifuged at 11,000× *g* for 15 min using a high-speed refrigerated centrifuge (H1850R, Hunan Xiangyi Laboratory Instrument Development Co., Ltd., Changsha, China) to isolate the complex precipitate. Subsequently, 0.1 g of the precipitate was weighed and dispersed in 100 mL of distilled water to a total volume of 200 mL in a beaker, followed by the slow addition of 1 mL of BEO. The Pickering emulsion was prepared by homogenizing at 14,000 rpm for 3 min using a high-speed homogenizer (FSH-2B, Changzhou Fangke Instrument Co., Ltd., Changzhou, China).

#### 2.2.3. Particle Size Distribution, Zeta Potential, and Polydispersity Index (PDI) of Nanoparticles and Pickering Emulsions

The particle size distribution, zeta potential, and PDI of the nanoparticles and Pickering emulsions were measured at 25 °C using a Nano ZSE analyzer (Malvern Instruments, Malvern, UK); the measurements of each treatment was performed in triplicate on three independently prepared samples, and the reported values represent the mean ± standard deviation of the replicates.

#### 2.2.4. Circular Dichroism (CD) Spectroscopy of Nanoparticles

CD spectra were acquired using a spectropolarimeter (J-815, JASCO, Tokyo, Japan) under continuous nitrogen purging in the far-ultraviolet region (190–250 nm). The secondary structure content, was analyzed and quantified using CDNN software (version 1.2, modified 9 October 1998; Gerald Böhm, Institute of Biochemistry and Biotechnology, Martin Luther University Halle-Wittenberg, Halle/Saale, Germany).

#### 2.2.5. Optical Microscopy Observation of PE

A small aliquot of the PE was deposited onto the center of a glass slide. The microstructure of the emulsions stabilized by SC and SC-TA particles was examined using optical microscopy (PA53 Bio, Motic Group Co., Ltd., Xiamen, China) under a 400× objective lens.

#### 2.2.6. Encapsulation Efficiency (EE) of PE

The encapsulation efficiency of the Pickering emulsions were determined based on the method described by Ran et al. [17] with slight modifications. To quantify BEO content, calibration standards were prepared in ethanol at seven distinct BEO concentrations (2–14 μg/mL, in 2 μg/mL increments). UV-Vis spectral analysis (TU-1901, Puyang Instrument Co., Puyang, China) was performed at 213 nm, yielding a linear regression model: y = 0.017x + 0.7596, with excellent correlation (R^2^ = 0.9991). For analytical processing, the Pickering emulsion sample (1 mL aliquot) was subjected to a 10-fold dilution with absolute ethanol followed by centrifugation (10 min, 5000× *g*). The absorbance of the supernatant was measured after appropriate dilution, and the concentration of free BEO was determined using the calibration curve. The measurements of each treatment were performed in triplicate on three independently prepared samples, and the reported values represent the mean ± standard deviation of the replicates. The encapsulation efficiency was calculated using the following Formula (1):

(1) $$EE(\%)\;={(C}_{0}\;-\;C_{1})/C_{0}\times \;100$$

where *C*_0_ represents the total concentration of bergamot essential oil (BEO) (μg/mL), and *C*_1_ denotes the concentration of free BEO in the Pickering emulsion (μg/mL).

#### 2.2.7. The Release of Essential Oil During Pickering Emulsion Storage

The essential oil Pickering emulsion was mixed with ethanol (1:9 *v*/*v*), and the mixture was shaken for 30 min. Then, the mixture was analyzed with an ultraviolet spectrophotometer (TU-1901, Puyang Instrument Co., Puyang, China) by measuring the absorbance of the Pickering emulsion at 213 nm. The release of essential oil was obtained as weight percentage of amount of BEO lost during storage to the initial amount of BEO in the Pickering emulsion. The measurements of each treatment was performed in triplicate on three independently prepared samples [18].

### 2.3. Preparation and Characterization of Carboxymethyl Tara Gum and Composite Coating Solution

#### 2.3.1. Preparation of Carboxymethyl Tara Gum

Following the methodology described by Barreto Santos et al. [7], 0.617 g of NaOH powder and 1.25 g of TG were mixed with 3 mL of 95% ethanol (C_2_H_5_OH) using a magnetic stirrer (SHJ-6AB, Changzhou Jintan Liangyou Instrument Co., Ltd., Changzhou, China) at 30 °C for 20 min. Then, 0.8022 g of MCA was added, and the reaction proceeded for 20 min. The product was kept at 60 °C for 10 h, dried at room temperature, dissolved in water, precipitated by ethanol, washed with 80% (*v*/*v*) methanol aqueous solution, then washed with absolute methanol, and finally dried at 45 °C. After dissolution in distilled water, the solution was subjected to dialysis for 48 h using an 8000 Da molecular weight cutoff (MWCO) dialysis membrane, followed by lyophilization using a vacuum freeze dryer (ZL-10N, Guangdong Zhongleng Refrigeration Technology Co., Ltd., Dongguan, China). Scheme 1b illustrates the chemical structure of TG before and after its carboxymethylation.

#### 2.3.2. Preparation of Composite Coating Solutions

1 g of carboxymethyl tara gum was completely dissolved in 40 mL of distilled water, after which 0.60 g of glycerol was added. The mixture was magnetically stirred at 800 rpm and 60 °C for 1 h to obtain a homogeneous CMTG-based coating solution. 1 g of TG was dissolved in 50 mL of distilled water, then 0.4 g of glycerol was added, and the mixture was stirred at 800 rpm and 60 °C for 1 h. The equal volume was mixed with Pickering emulsion and stirred together for 30 min, which was named TG + PE. The CMTG coating solution was mixed with Pickering emulsion and stirred together for 30 min, which was named CMTG + PE. The coating solution was dried into a film for testing when necessary. The solution was uniformly poured on a polystyrene Petri dish and dried at room temperature for 48 h to form films, which were then subjected to testing. Since PE alone could not form a stable film, the PE group was excluded from certain tests. The simple preparation flowchart is shown in Scheme 1c.

#### 2.3.3. Scanning Electron Microscopy (SEM)

The dried film was mounted on conductive adhesive, and the surface and cross-sectional morphology were examined using a cold field-emission scanning electron microscope (JSM-7500F, JEOL Ltd., Tokyo, Japan) after sputter coating with platinum. The accelerating voltage was set to 5 kV.

#### 2.3.4. Fourier Transform Infrared (FTIR) Spectroscopy

Fourier transform infrared spectroscopy (Is5, Thermo Fisher, Waltham, MA, USA) was used to analyze the samples within the wavenumber range of 4000–500 cm^−1^ at a resolution of 4 cm^−1^.

#### 2.3.5. Thermal Stability

The thermal stability of the film was evaluated using a thermogravimetric analyzer (TGA/DSC 1/1600HT, Mettler-Toledo, Greifensee, Switzerland), with the temperature ramped from 25 °C to 800 °C at a heating rate of 10 °C/min.

#### 2.3.6. Film Thickness Measurement

Film thickness quantification was measured using a digital micrometer, where multiple measurements (*n* ≥ 3) were taken at random locations across each specimen to obtain a representative mean thickness value.

#### 2.3.7. Water Vapor Permeability (WVP)

The WVP of each film was determined according to the method of Xie et al. [19]. Each group comprised three films cast from the same mold with identical formulation. These films were cut to the same effective area and individually sealed onto glass containers filled with anhydrous calcium chloride, ensuring that the distance between the desiccant surface and the container opening remained below 6 mm. The assembly was placed in a desiccator containing saturated sodium chloride solution, and the weight was recorded at 12 h intervals until equilibrium was reached. The WVP was calculated using the following Formula (2):

(2) WVP(g/Pa·h·m)=(Δw×D)/(Δt×A×Δp)

where Δ*w*/Δt represents the rate of weight gain per hour (g/h), A denotes the exposed area of the film (m^2^), *D* corresponds to the film thickness (m), and Δp indicates the water vapor pressure gradient across the film.

#### 2.3.8. Mechanical Properties

The mechanical property tests were conducted according to the modified Chen et al. [20]: Each group independently prepared three identical films, and each film was cut into 1 cm × 7 cm strips, and analyzed using a texture analyzer (TA.XT Plus, Stable Micro Systems, Godalming, UK). The tensile strength (TS) and elongation at break (EAB) were calculated using the following Formulas (3) and (4):

(3) TS(MPa) = F/(T × W)

(4) $$E\mathrm{AB}(\%)=(L\;-\;L_{0})/L_{0}\;\times \;100$$

where *F* stands for the maximum force (N), *T* and W correspond to the thickness (mm) and width (mm) of the sample, respectively, *L* corresponds to the final length at rupture, and *L*_0_ indicates the initial gauge length of the film between the clamps.

#### 2.3.9. Antimicrobial Activity

Following the methodology described by Yusufe Adame et al. [21] with modifications, the antimicrobial activity of the coating solution against *Escherichia coli* and *Staphylococcus aureus* was evaluated. A 100 μL aliquot of the bacterial suspension (10^8^–10^9^ CFU/mL) was inoculated onto the surface of the agar medium. A sterile filter paper disk with a diameter of 6 mm was placed at the center of the agar medium, and the coating solution was applied to the disk. The inhibition zones were measured after incubation at 37 °C for 24 h. Each tablet was measured once, resulting in 3 independent data points. The results were expressed as the average value ± standard deviation of three technical repetitions (*n* = 3).

#### 2.3.10. Antioxidant Activity

The DPPH and ABTS radical-scavenging assays were conducted according to the modified method of Wang et al. [22]. For each assay, three independent reaction mixtures were prepared, and absorbance was measured at 517 nm (DPPH) or 734 nm (ABTS).

DPPH free radical scavenging activity: Equivalent volumes of the coating formulation and 0.1 mM DPPH ethanolic solution were combined. The reaction mixture was maintained under dark conditions at ambient temperature (25 ± 2 °C) for 30 min before spectrophotometric analysis at 517 nm wavelength.

ABTS free radical scavenging activity: The ABTS radical scavenging assay was performed by combining 2 mL of the coating solution with 3 mL of ABTS reagent, followed by incubation at physiological temperature (37 °C) for 30 min. The resulting solution was then analyzed spectrophotometrically at 734 nm. The scavenging activity was calculated using the following Formula (5):

(5) $$\mathrm{DPPH}/\mathrm{ABTS}\;\mathrm{scavenging}\;\mathrm{activity}(\%)\;=\;(A_{0}\;-(A\;-\;A_{C}))/A_{0}\times \;100$$

where the blank group (*A*_0_) consisted of a mixture of ethanol/distilled water and DPPH/ABTS solution, the sample group (*A*) represented a mixture of the coating solution and DPPH/ABTS solution, and the control group (*A_C_*) comprised a mixture of the coating solution and ethanol/distilled water.

### 2.4. Evaluation of the Preservation Efficacy of Coatings on Cherry Tomatoes

#### 2.4.1. Coating Treatment of Cherry Tomatoes

Cherry tomatoes with intact appearance, uniform size, and color, and free from pests, diseases, and mechanical damage were selected. For effective surface decontamination, the chosen cherry tomatoes underwent dual sequential treatments, each involving a 3 min immersion in 2% (*w*/*v*) NaOCl disinfectant solution, with thorough rinsing between applications to ensure complete sanitization. Afterwards, sterile 2 mm perforations were made in each tomato specimen using a biopsy punch under laminar airflow. Following air-drying, a standardized 10 μL aliquot of *Streptomyces* sp. inoculum (1 × 10^6^ colony-forming units/mL) was aseptically delivered into each wound site using a micropipette, then allowed to air-dry under ambient laboratory conditions (25 ± 2 °C, 60% RH). The fruits were then immersed in the composite coating solution for 3 min and air-dried, with distilled water used as the control treatment. Finally, the fruits were stored at 25 ± 1 °C with a relative humidity of 80–90% and randomly divided into three groups: Control, CMTG, and CMTG + PE. Each treatment group had 100 cherry tomatoes, making a total of 300 cherry tomatoes. The fruits were stored for 8 days, and physicochemical properties were measured and recorded at 0, 2, 4, 6, and 8 days.

#### 2.4.2. Sensory Analysis of Cherry Tomatoes on the 8th Day of Storage

On the 8th day of the storage period, ensuring location and light conditions, a panel of 6 members (3 females and 3 males, aged 20–40 years) scored tests (on a five-point scale) on the color, odor and consumer acceptance of cherry tomatoes coated with three groups of films (1 = the most unacceptable degree, 5 = the most acceptable degree) [23].

#### 2.4.3. Weight Loss, Firmness, and Respiration Rate

The cherry tomatoes were weighed at 0, 2, 4, 6, and 8 days. Place 4 cherry tomatoes in the same polyethylene open box and directly weigh the entire box of cherry tomatoes. Each treatment group prepared three boxes of cherry tomatoes for weighing, and the weight loss percentage was determined according to Formula (6):

(6) $$\mathrm{Weight}\;\mathrm{loss}(\%)\;=\;(W_{0}-W_{1})/W_{0}\;\times \;100$$

where *W*_0_ represents the initial weight, and *W*_1_ denotes the weight at a specific storage time.

The textural properties of cherry tomato samples were quantitatively assessed through instrumental texture analysis (TA.XT Plus, Stable Micro Systems, Godalming, UK). A P/2 probe was employed to penetrate at the equator of the fruit sample to a depth of 5 mm. For each treatment, three fruits were analyzed daily, with three replicate measurements taken per fruit.

Approximately 80 g of cherry tomatoes were placed in a 500 mL beaker, sealed with plastic wrap, and equilibrated at 25 °C for 20 min. Each treatment group prepared three portions of cherry tomatoes. The CO_2_ concentration within the beaker was measured using a C_2_H_4_/O_2_/CO_2_ analyzer probe (F-940, Yangguangyishida, Beijing, China). Respiration rate was expressed as the mass of CO_2_ (mg) produced per kilogram of fresh weight per hour.

#### 2.4.4. Total Soluble Solids (TSS) and Titratable Acidity

TSS content was measured by applying 0.3 mL of freshly extracted cherry tomato juice onto the prism surface of a digital refractometer (PAL-1, ATAGO, Tokyo, Japan) at 20 °C, with results expressed as Brix. Each treatment group was measured using three different cherry tomatoes every day.

Precisely weighed cherry tomato tissue (10.0 ± 0.1 g) was quantitatively transferred to a 100 mL volumetric flask and brought to volume with deionized water using a graduated pipette for final mixing. After allowing the mixture to stand for 30 min, 20 mL of the filtrate was mixed with phenolphthalein indicator and titrated with 0.1 M sodium hydroxide solution [24]. For each treatment group, three different samples of cherry tomato powder were used for each measurement. Titratable acidity was calculated using the following Formula (7):

(7) $$\mathrm{Titratable}\;\mathrm{acidity}\;\mathrm{content}(\%)\;=\;(V\times c\times \left(V_{1}-V_{0}\right)\;\times \;f)/(V_{S\;}\times \;m)\times 100\%$$

where *V* represents the total volume of the sample extract (mL), *Vₛ* denotes the volume of filtrate used for titration (mL), c indicates the concentration of the NaOH titrant (mol/L), *V*_1_ corresponds to the volume of NaOH solution consumed during titration of the filtrate (mL), *V*_0_ signifies the volume of NaOH solution consumed during titration of distilled water (mL), *m* is the sample mass (g), and f is the conversion factor (g/mmol).

#### 2.4.5. Vitamin C (VC) and Lycopene

A 10 g sample of cherry tomatoes was homogenized with 20 g/L oxalic acid. The resulting slurry was quantitatively transferred to a 100 mL volumetric flask and brought to volume with the same oxalic acid solution. After allowing the mixture to stand for 10 min, 10 mL of the filtrate was titrated with 2,6-dichlorophenol indophenol solution [25]. For each treatment group, three different samples of cherry tomato powder were used for each measurement. The VC content was formulated as Formula (8):

(8) $$\mathrm{Vitamin}\;\mathrm{content}(\mathrm{mg}/100\;g)\;=\;(V\times (V_{1}-V_{0})\times \rho )/{(V}_{S}\times m)\times 100$$

where *V*_1_ represents the volume of 2,6-dichlorophenol indophenol solution consumed during sample titration (mL), V_0_ denotes the volume of 2,6-dichlorophenol indophenol solution consumed during blank titration (mL), ρ indicates the mass of vitamin C equivalent to 1 mL of 2,6-dichlorophenol indophenol solution (mg/mL), vs. corresponds to the volume of sample solution used for titration (mL), *V* is the total volume of the sample extract (mL), and *m* is the mass of the sample (g).

The method described by Muzolf-Panek et al. [26] was adapted with minor modifications. A 2 g sample of cherry tomato powder was added to a conical flask, followed by the addition of 5 mL of ethanol and 40 mL of petroleum ether. The mixture was thoroughly mixed, and the flask was sealed. The extraction was performed under photoprotection at 4 ± 1 °C for 40 min. Following phase separation, the supernatant was immediately analyzed spectrophotometrically at 503 nm. The measuring result was compared with the standard sample of known lycopene concentration. For each treatment group, three different samples of cherry tomato powder were used for each measurement. Using the calibration curve equation y = 0.1319x − 0.0781 (R^2^ = 0.9995), the lycopene content was calculated using the following Formula (9):

(9) $$\mathrm{Lycopene}\;\mathrm{content}\;(\mu g/g\;\mathrm{FW})=(A_{503}/0.1635\times n)/m$$

where n represents the dilution factor, and *m* denotes the sample mass.

#### 2.4.6. Malondialdehyde (MDA) Concentration

A 1 g sample of cherry tomato powder was weighed, and 5 mL of 10% (*w*/*v*) trichloroacetic acid (TCA) solution was added. After homogenization, the mixture was centrifuged at 10,000× *g* and 4 °C for 20 min. A precisely measured 2.00 mL portion of the clarified supernatant was combined with an equal volume of 0.67% (*w*/*v*) thiobarbituric acid reagent in a test tube. The mixture was heated in a boiling water bath for 20 min, cooled, and centrifuged again (For each treatment group, three different samples of cherry tomato powder were used for each measurement.). After centrifugation, the absorbance values of the supernatant at 450, 532, and 600 nm were measured using an UV–Vis spectrophotometer (TU-1901, Puyang Instrument Co., Puyang, China), with subsequent quantification via the established Formula (10):

(10) $$\mathrm{MDA}\;\mathrm{content}\;(\mu \mathrm{mol}/g\;\mathrm{FW})\;=\;(c\;\times \;V)/{(V}_{S}\;\times \;m\;\times \;1000)$$

where c represents the malondialdehyde (MDA) concentration in the mixture, calculated as c (μmol/L) = 6.45 × (OD_532_ − OD_600_) − 0.56 × OD_450_; V denotes the total volume of the sample extract (mL), *Vₛ* corresponds to the volume of the sample aliquot used for analysis (mL), and *m* is the sample mass (g).

### 2.5. Statistical Analysis

Triplicate independent experiments were conducted for each treatment condition. Numerical datasets were processed and visualized through Origin 2024 software, with inferential statistics performed using SPSS 26.0 (IBM). Significant intergroup differences (*p* < 0.05) were identified via ANOVA coupled with Duncan’s multiple comparison procedure.

## 3. Results and Discussion

### 3.1. Characterization of Nanoparticles and Pickering Emulsions

#### 3.1.1. FTIR Spectroscopic Analysis of Nanoparticles

**Figure 1:**
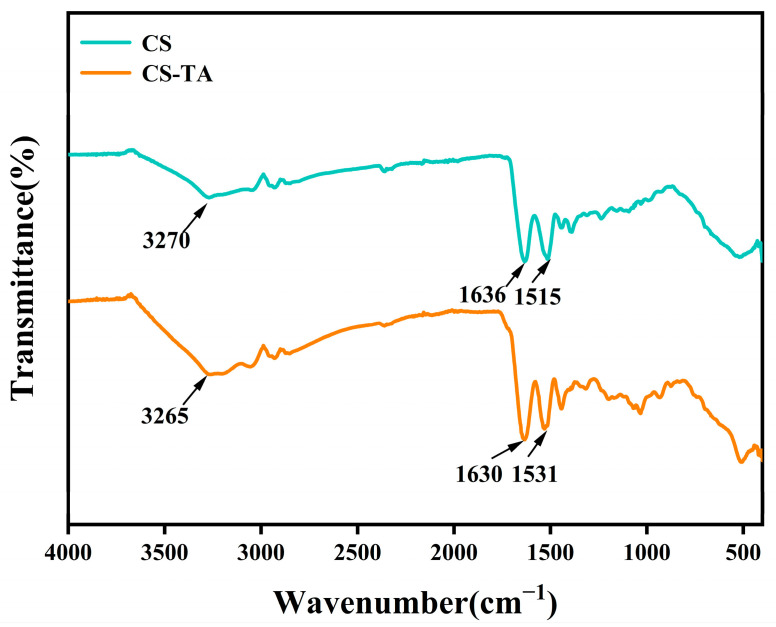
FTIR spectra of solid particles.

FTIR spectroscopy can elucidate the interactions between SC and TA. As shown in Figure 1, the absorption peak at 3271–3273 cm^−1^ corresponds to the stretching vibrations of N–H and O–H bonds, and the peak at 1630–1636 cm^−1^ is attributed to amide I, primarily resulting from the C = O stretching vibration, and the peak at 1514–1526 cm^−1^ is associated with amide II. This phenomenon primarily results from coupled vibrational modes involving N-H bond deformation and C-N bond elongation [27]. FTIR spectral analysis of SC-TA complexes revealed significant band shifts compared to native SC. In contrast, the amide II peak displayed a blueshift of 12 cm^−1^, likely due to the formation of hydrogen bonding interactions between SC and TA [28]. Additionally, an increase in peak intensity at amide I was observed for SC-TA, likely resulting from the binding of tannic acid (TA) to the C = O groups of the protein (hydrophilic interaction) [29].

#### 3.1.2. CD Spectroscopic Analysis of Nanoparticles

CD spectroscopic analysis was performed to examine the conformational changes induced in SC upon interaction with TA. As summarized in Table 1, the secondary structure of untreated SC consisted of 35.93% α-helix, 0.70% β-sheet, 31.62% β-turn, and 31.75% random coil. Structural analysis revealed significant secondary structure alterations upon TA treatment, with an elevation in β-sheet conformation accompanied by reductions in both β-turn structures and unordered random coil elements. These findings are consistent with the results reported by Jia et al. [30], suggesting that the secondary structure of SC is altered following TA modification. The observed conformational rearrangement likely stems from TA-protein hydrogen bonding interactions, which induce secondary structure transitions toward β-sheet configurations. This molecular restructuring leads to polypeptide chain extension and subsequent stabilization of the SC matrix.

#### 3.1.3. Particle Size Distribution, Zeta Potential, and PDI of Nanoparticles and Pickering Emulsions

The physicochemical characteristics of both nanoformulations and their corresponding Pickering systems, including Particle size distribution, zeta potential, and PDI, are presented in Table 2. The results indicate that the addition of TA did not significantly affect the particle size of SC, likely due to the small size of TA particles and the small amount added, allowing the nanocomplex to retain the size characteristics of SC. With the incorporation of TA, the negative charge of the nano complex increased, likely due to the protonation of TA and the generation of oxygen centers, which impart a high negative charge density to SC, enhancing its dispersion stability in aqueous solutions [16]. The PDI of pure SC was 0.4, whereas that of the SC-TA nanocomposite was 0.64, suggesting that TA did not improve the dispersion stability of SC in aqueous solutions. Smaller droplet sizes result in greater dispersion and a higher specific surface area [31]. A lower PDI value indicates a narrower size distribution, while a higher absolute zeta potential suggests a more stable emulsion system [32]. The particle size of the Pickering emulsion stabilized by the SC-TA nanocomposite were lower than those of the emulsion stabilized by SC alone, and the absolute zeta potential increased, indicating that the stabilization capacity of SC for Pickering emulsions was enhanced after TA treatment. This phenomenon may be attributed to the strong electrostatic interactions between highly charged solid particles, which restricts their coagulation or flocculation. This interaction enhance the interfacial film strength and stability of the Pickering emulsion [33].

#### 3.1.4. Microstructural Characterization of Pickering Emulsions and Encapsulation Efficiency

**Figure 2:**
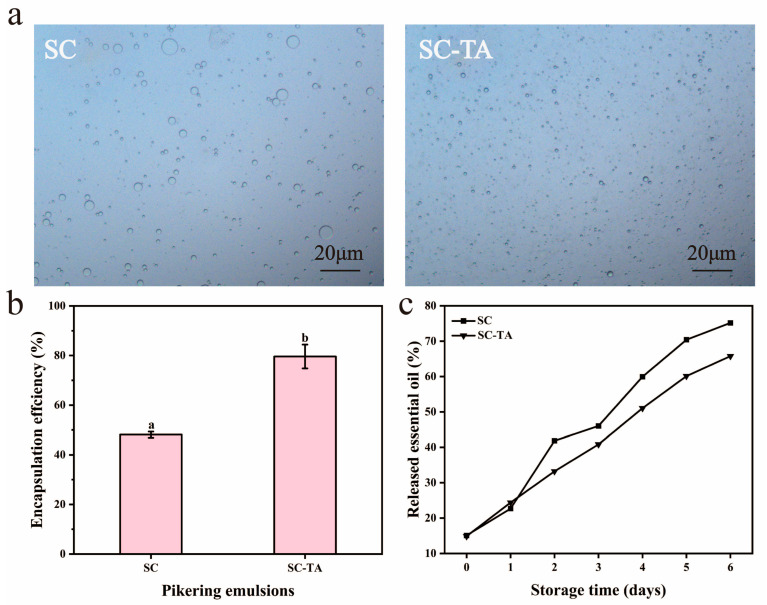
Characterization of the Pickering emulsions. (**a**) Microstructural analysis of the Pickering emulsions. (**b**). Encapsulation efficiency the Pickering emulsions. Different lowercase letters indicate significant difference (*p* < 0.05). (**c**) The sustained-release rate of Pickering emulsion.

Figure 2a illustrates the microstructure of Pickering emulsions stabilized by SC and SC-TA nanocomposites, respectively. It is evident that while both Pickering emulsions exhibit a regular spherical morphology, the droplet size distribution of the SC-stabilized emulsion is heterogeneous, with a relatively dispersed spatial arrangement. However, the droplets of the SC-TA-stabilized Pickering emulsion were significantly smaller, with a denser spatial distribution and more uniform droplet size. This phenomenon may be attributed to the higher negative charge density of the modified nanocomplex, which enhances the electrostatic interactions between droplets, leading to a more uniform distribution of the Pickering emulsion.

Figure 2b demonstrates significantly higher encapsulation efficiency (*p* < 0.05) for the TA-modified SC emulsion system (79.62 ± 4.77%) compared to the native SC-stabilized counterpart (48.13 ± 1.24%). The encapsulation efficiency serves as an indicator of emulsion stability, with higher values corresponding to greater stability [34]. The results demonstrate that TA modification enhanced BEO incorporation into the emulsion. This improvement may be attributed to the formation of a denser and more cohesive interfacial layer through SC–TA complexation, which enhances adsorption at the oil-water interface and reduces droplet coalescence. The improved interfacial film strength and stability imparted by SC-TA to the Pickering emulsion thus contribute to higher encapsulation efficiency.

#### 3.1.5. The Sustained-Release Rate of Pickering Emulsion

As can be seen from Figure 2c, the release rate of essential oil from the Pickering emulsion with stable SC is relatively unstable. However, the release rate of essential oil from the Pickering emulsion stabilized by the SC-TA nanocomposite is relatively stable and slower than that stabilized by SC. This may be attributed to the high negative charge of the SC-TA nanocomposite, which generates strong electrostatic repulsion, inhibits the aggregation of interface particles, and reduces the leakage of essential oil caused by emulsion flocculation. This result is consistent with the zeta potential of solid particles.

### 3.2. Characterization of CMTG and Composite Coating Formulations

#### 3.2.1. SEM

**Figure 3:**
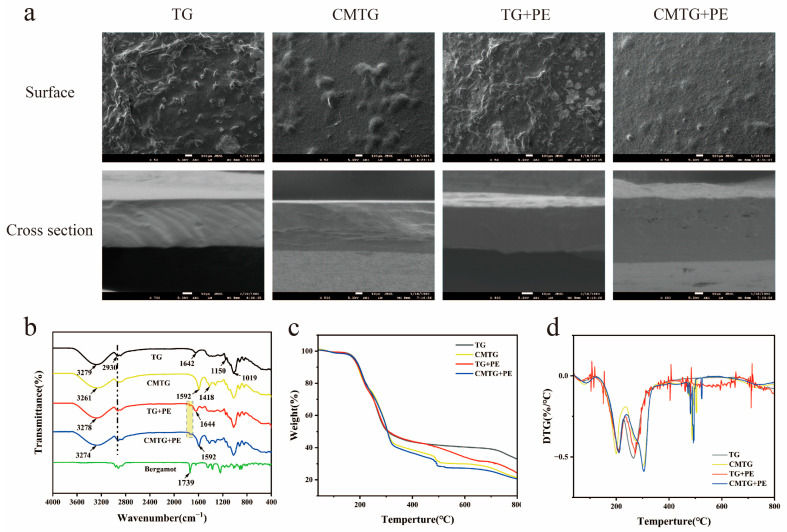
Characterization of the films. (**a**) SEM images of the film surface and cross-section. (**b**) FTIR spectra of the films. (**c**) TGA curves of the films. (**d**) DTG of the films.

SEM was used to examine the surface morphology and cross-sectional density of the films. As shown in Figure 3a, the surface of the CMTG film exhibited significantly smoother and less rough features compared to the unmodified TG film. Furthermore, the cross-sectional analysis revealed that the modification preserved the dense structure characteristic of the TG film. The surface of the film incorporating PE displayed uniformly distributed spherical protrusions, likely resulting from the migration of emulsion droplets to the surface during film drying [32]. In contrast, the surface of the CMTG + PE film appeared more refined, suggesting enhanced compatibility between PE and CMTG compared to unmodified TG. This observation is further supported by the presence of voids within the cross-section of the CMTG + PE film, resulting from the evaporation of essential oils under vacuum conditions.

#### 3.2.2. FTIR Spectroscopy of the Films

The FTIR spectral profile of TG (Figure 3b) revealed characteristic vibrational bands at 3261–3279 cm^−1^, indicative of O-H stretching in galactose/arabinose residues, and a peak at 2930 cm^−1^, corresponding to the C–H stretching vibration of alkyl groups. The 1642 cm^−1^ absorption band corresponds to carboxylate (COO^−^) asymmetric stretching vibrations [35], whereas the adjacent spectral features at 1019 cm^−1^ and 1050 cm^−1^ arise from glycosidic linkage C-O stretching and the C–O bending vibration of pyranosyl rings, respectively [36]. The CMTG spectrum displayed characteristic carboxylate vibrations, with antisymmetric stretching at 1592 cm^−1^ and symmetric stretching at 1418 cm^−1^, confirming successful carboxymethyl modification through these diagnostic infrared bands [37]. And the FTIR analysis revealed a novel absorption band at 1793 cm^−1^ in both TG + PE and CMTG + PE composites, characteristic of ester carbonyl stretching vibrations from BEO constituents [38]. This spectroscopic marker confirms successful integration of the Pickering emulsion into the polymeric matrix.

#### 3.2.3. Thermal Stability

Thermal stability and degradation of the films were evaluated through thermogravimetric analysis (TGA, Figure 3c) and derivative thermogravimetric analysis (DTG, Figure 3d). The thermal degradation of TG and CMTG films primarily occurs in three stages. The initial mass loss event (25–170 °C) represents the endothermic elimination of physisorbed water molecules from the material’s surface and porous structure. The second stage, between 200 and 300 °C, involves the degradation of glycerol as a plasticizer and the majority of polysaccharides. The third stage, above 300 °C, is attributed to the degradation of residual polysaccharides and the carbonization and combustion of other components [39,40]. Following the incorporation of PE, the residual mass of the films within the 200–300 °C range was higher compared to films without PE, indicating that PE impeded the thermal decomposition of the films and enhanced their thermal stability [41]. Furthermore, the maximum thermal degradation temperatures of TG, CMTG, TG + PE and CMTG + PE were 266.0 °C, 302.4 °C, 272.2 °C and 305.6 °C, respectively, indicating that both the carboxymethylation of TG and the addition of PE enhanced the thermal stability of the composite membrane.

#### 3.2.4. WVP

WVP is a critical parameter for evaluating the effectiveness of films in preserving the quality of fruits and vegetables by minimizing water vapor transmission. Table 3 demonstrates a 21.43% reduction in WVP for the CMTG film relative to the native TG matrix, correlating with the 39.22% increase in measured thickness (51.00 ± 1.00 μm vs. 71.00 ± 2.00 μm). The incorporation of PE did not significantly alter the WVP of TG films (*p* > 0.05). This phenomenon likely stems from (i) insufficient interfacial compatibility between the TG matrix and PE droplets, and (ii) surface migration of emulsion particles during solvent evaporation, leading to partial loss of volatile components—as corroborated by SEM surface topography analysis. However, when CMTG is combined with PE, the WVP is significantly reduced, likely due to the effective embedding of Pickering emulsion droplets within the CMTG matrix, forming a denser network structure that restricts water vapor migration. These findings are consistent with those reported by Zhikun Yang et al. [42], who observed a similar reduction in WVP for composite films incorporating baobab seed oil Pickering emulsion.

#### 3.2.5. Mechanical Properties

CMTG films demonstrated significant mechanical improvements compared to those of TG films (Table 3), with tensile strength (TS) increasing from 4.81 ± 0.81 MPa to 11.39 ± 0.29 MPa and elongation at break (EAB) rising from 19.22 ± 3.83% to 40.89 ± 11.57%, may attributable to newly established electrostatic interactions and hydrogen bonding networks (As illustrated in Figure 3b, the CMTG film exhibits an appreciable broadening of the ν (O–H) envelope spanning 3200–3600 cm^−1^, accompanied by a distinct red-shift relative to the native TG film.) [43]. The incorporation of PE had contrasting effects on the TS of TG and CMTG films, indicating that PE negatively impacted the tensile strength of TG films but improved that of CMTG films. This result further suggests the formation of chemical bonds, such as hydrogen bonds, between PE and CMTG, leading to a denser structure. However, the EAB of both TG and CMTG films decreased after the addition of PE, likely due to the transition from a continuous to an irregular film structure, which is consistent with the findings reported by Ming Cheng et al. [34].

#### 3.2.6. Antimicrobial Activity

**Figure 4:**
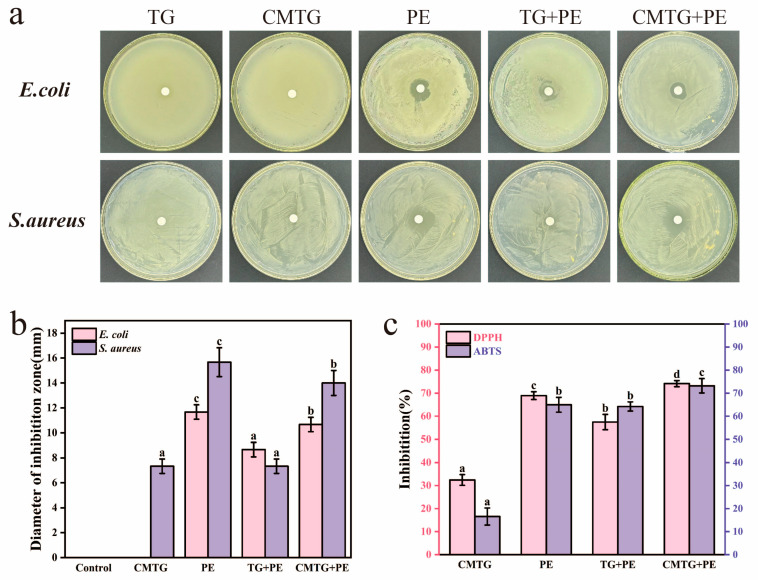
(**a**) Antimicrobial activity of the films. (**b**) The antibacterial zone of the films. (**c**) Antioxidant activity of the films. Different lowercase letters indicate significant difference (*p* < 0.05).

Fruits are highly susceptible to microbial infection and spoilage, so evaluating the antimicrobial activity of coating solutions is critically important. As shown in Figure 4a, CMTG exhibited slight inhibitory activity against *Staphylococcus aureus*, while the PE demonstrated significant antimicrobial effects against both *Escherichia coli* and *Staphylococcus aureus*, with inhibition zones exceeding 10 mm (Figure 4b). After adding it to the film-forming solution, the antibacterial zone slightly decreased. This is because the addition process would dilute the concentration of PE. However, the CMTG + PE coating solution can still effectively inhibit bacteria. This is attributed to the ability of the primary components of BEO, D-limonene, and γ-terpinene, to disrupt microbial cell membranes, inhibit respiratory enzyme activity, and dissipate proton motive force, thereby exerting antimicrobial effects [10].

#### 3.2.7. Antioxidant Activity

Free radicals can degrade food quality and compromise its edibility, making free radical scavenging activity in coating solutions highly significant. The PE incorporation markedly improved the antioxidant capacity of coating formulations. Specifically, the CMTG + PE formulation increased the DPPH and ABTS scavenging rates by approximately 42% and 57% (Figure 4c), respectively, compared to CMTG alone. This improvement is likely attributed to the presence of various antioxidant components in BEO, such as terpenes, phenylpropanoids, aldehydes, esters, alcohols, and ketones [44], which exhibit potent free radical scavenging properties. Additionally, CMTG demonstrated superior radical scavenging activity against DPPH radicals (32.43 ± 2.33%) compared to ABTS^+^ (16.57 ± 3.71%), likely resulting from increased electron density at oxygen atoms introduced through carboxymethyl substitution. This electronic configuration preferentially facilitates hydrogen atom transfer to DPPH radicals. These findings align with the results reported by Rehebati Nuerxiati et al. [45].

### 3.3. Evaluation of the Preservation Efficacy of Coatings on Cherry Tomatoes

#### 3.3.1. The Visual Appearance of Cherry Tomatoes and the Sensory Analysis of Cherry Tomatoes on the 8th Day of Storage

**Figure 5:**
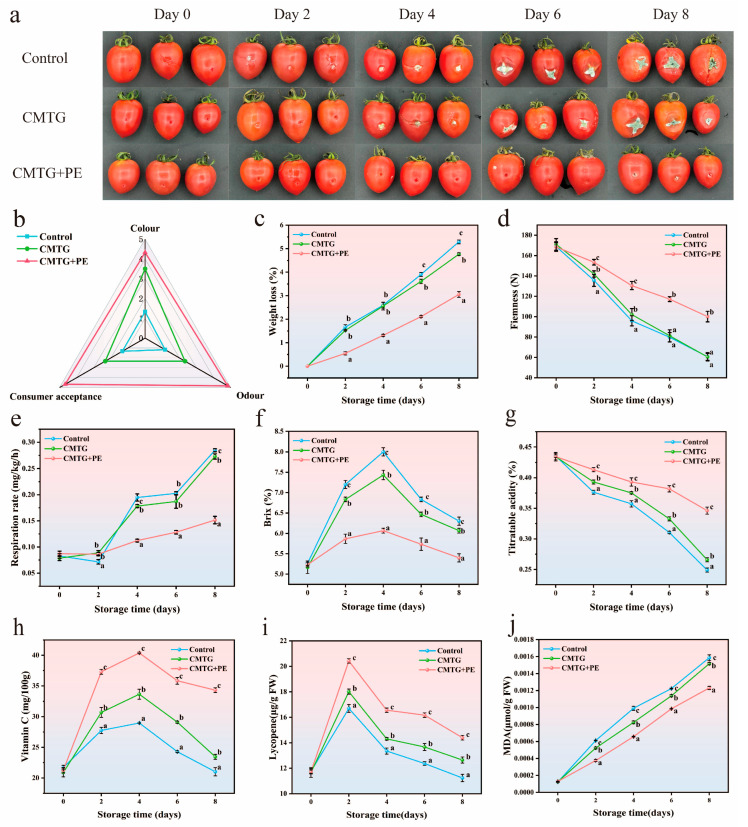
(**a**) The visual appearance of cherry tomatoes. (**b**) Sensory analysis of cherry tomatoes on the 8th day of storage. (**c**) Weight loss changes in cherry tomatoes. (**d**) Firmness changes in cherry tomatoes. (**e**) Respiration rate changes in cherry tomatoes. (**f**) TSS content changes in cherry tomatoes. (**g**) TA content changes in cherry tomatoes. (**h**) Changes in VC content in cherry tomatoes. (**i**) Changes in lycopene content in cherry tomatoes. (**j**) Changes in MDA content in cherry tomatoes. Different lowercase letters indicate significant difference (*p* < 0.05).

The visual appearance of fruits is closely linked to their quality and significantly influences consumer acceptance. As shown in Figure 5a, control samples developed visible mycelial networks at wound sites, while coated specimens maintained complete surface sterility. Subsequently, all groups except the CMTG + PE group displayed extensive mycelial proliferation, accompanied by softening and shrinkage. In contrast, the CMTG + PE group showed delayed mycelial growth, which only became apparent after the sixth day, while maintaining a plump and smooth appearance until the eighth day. Referring to Figure 5b, the results show the sensory scores given by the professionals for the cherry tomatoes collected on the 8th day of storage. The scores of the CMTG + PE coating group in all three aspects were higher than those of the other two groups. This preservation effect is attributed to the coating’s ability to isolate the cherry tomatoes from the external environment, thereby slowing oxidative and decay processes, while the controlled release of BEO further inhibits mycelial development. This study demonstrates a notably enhanced preservation effect compared to the work by Yu et al. [46]. They used hydroxypropyl methylcellulose and maltodextrin to make coatings for preserving cherry tomatoes inoculated with *Alternaria alternata*, but the cherry tomatoes they stored could show visible fungal hyphae growing inside the holes on the second day, while in this study, only a small amount of fungal hyphae could be observed growing on the sixth day.

#### 3.3.2. Weight Loss, Firmness, and Respiration Rate

The weight of fruits and vegetables decreases due to the loss of water and nutrients, making weight loss a critical parameter for evaluating storage quality. Figure 5c demonstrates progressive mass reduction in all test groups throughout storage. Uncoated controls exhibited the highest weight loss rate (5.29 ± 0.12% by day 8), whereas CMTG and CMTG + PE coatings significantly (*p* < 0.05) reduced dehydration to 4.76 ± 0.09% and 3.05 ± 0.07%, respectively, through modified atmospheric protection. This difference may result from the thicker coating and lower WVP of the CMTG + PE formulation, which more effectively reduced gas exchange and transpiration between the fruit and the external environment. This mitigation is not only due to the physical barrier limiting gas exchange and water transpiration but may also linked to reduced respiratory metabolism and enzymatic activity, which collectively slow down metabolic water loss and substrate consumption.

Texture profile analysis reveals that compressive resistance, commonly measured as firmness, serves as a critical physicochemical parameter for evaluating postharvest fruit quality and freshness retention. As presented in Figure 5d, during storage, cherry tomatoes exhibited progressive textural softening (*p* < 0.05), primarily mediated through enzymatic hydrolysis of pectin polysaccharides by polygalacturonase and pectin methylesteras [47]. By the eighth day, the firmness of the CMTG + PE group was 100.07 N, representing a 40% increase compared to the control group. This demonstrates that the CMTG + PE composite coating significantly (*p* < 0.05) attenuated textural degradation. This protective effect arises from the dual functionality of BEO: (i) suppressing microbial pectinase production and (ii) maintaining cell wall integrity through antioxidant-mediated polysaccharide stabilization.

Fruits maintain a certain respiration rate during storage, which directly influences their shelf life. As demonstrated in Figure 5e, from the fourth day onward, the respiration rate of all groups exhibited a gradual increase, likely due to the accumulation of oxygen, metabolic intermediates, and enzymes, as well as pathogen-induced stress, which collectively elevated respiratory activity [48]. After the fourth day, the respiration rate of the coated groups was lower than that of the control group, with the CMTG + PE coating demonstrating the greatest inhibition of respiration. This effect is attributed to the synergistic action of the coating’s barrier properties and antimicrobial activity. The coating acts as a modified atmosphere barrier, restricting oxygen diffusion into the fruit tissue and consequently limiting the substrate availability for oxidative metabolic pathways, which suppresses the respiration rate. Moreover, the antimicrobial activity reduces the population of spoilage microorganisms, thereby diminishing the biotic stress that triggers defensive respiratory bursts in the fruit. The superior performance of the CMTG + PE coating likely stems from its optimal film-forming ability, creating a more efficient gas barrier, and its sustained release of antimicrobial agents, which provides prolonged protection against microbial provocation., the antimicrobial activity reduces the population of spoilage microorganisms, thereby diminishing the biotic stress that triggers defensive respiratory bursts in the fruit. The superior performance of the CMTG + PE coating likely stems from its optimal film-forming ability, creating a more efficient gas barrier, and its sustained release of antimicrobial agents, which provides prolonged protection against microbial provocation.

#### 3.3.3. TSS and Titratable Acidity

TSS is a critical parameter influencing fruit sweetness. As depicted in Figure 5f, the TSS exhibited a biphasic trend during storage, with initial elevation (Days 0–4) followed by gradual decline (Days 4–8). This pattern reflects pectinase-mediated hydrolysis of cell wall polysaccharides into reducing sugars, subsequently metabolized by fermentative pathways. Subsequently, fungal infection and nutrient loss in cherry tomatoes led to a decline in TSS during later stages [49]. The variation in TSS content was significantly lower in the coated groups compared to the control group, with the CMTG + PE group exhibiting the smallest variation. This is likely attributed to the coating’s ability to reduce respiration rates and metabolic activity, thereby minimizing TSS depletion.

Titratable acidity is closely associated with the organic acid content of fruits and significantly influences their acidity. As illustrated in Figure 5g, storage duration negatively correlated with titratable acidity levels, as organic acids were metabolized into sugars or consumed in mitochondrial respiration [50]. The reduction in titratable acidity was significantly smaller in the coated groups compared to the control group, with the CMTG + PE group maintaining significantly higher titratable acidity levels after the sixth day. This preservation effect may result from the antioxidant properties of the composite coating, which reduce oxidative and metabolic activity, thereby slowing the degradation of titratable acidity.

#### 3.3.4. VC and Lycopene

VC is a critical parameter for assessing the antioxidant capacity and nutritional quality of fruits. As shown in Figure 5h, during the early storage period, the VC content increased with the ripening of cherry tomatoes, with the CMTG + PE group exhibiting the highest increase. However, after the fourth day, the VC content began to decline, likely due to respiratory activity and microbial infection [51]. The CMTG + PE group showed the smallest reduction in VC content, which may be attributed to the superior barrier properties of the CMTG + PE film, which limited oxygen exposure, thereby slowing respiration and the degradation of organic acids.

Lycopene is the main pigment that gives cherry tomatoes their deep red color, and it is also an antioxidant. As evidenced by Figure 5i the lycopene content of cherry tomatoes in all treatment groups showed an increasing trend at the initial storage stage, which may be due to the increase in lycopene content in cherry tomatoes as they matured, but the lycopene content began to decrease from the 4th day, which may be due to the loss of nutrients in cherry tomatoes due to microbial infection. However, the content of lycopene in the coating group was significantly higher than that in the control group, and the decrease in lycopene in the CMTG + PE group was the slowest, which was consistent with the findings of Hafiz Muhammad Saleem Akhtar et al. [52].

#### 3.3.5. MDA

MDA content is a key indicator of lipid peroxidation in the cell membranes of fruits and vegetables. As revealed in Figure 5j, MDA levels exhibited a time-dependent increase in all treatment groups, likely due to the accumulation of MDA resulting from aging and deterioration during storage [53]. However, the MDA content in the coated groups was significantly lower than that in the control group after the second day (*p* < 0.05), with the CMTG + PE group showing the least accumulation and variation. This reduction can be attributed to the coating’s ability to mitigate oxidative injury through two main mechanisms: (1) by acting as a barrier that reduces oxygen availability, thereby limiting the initiation and propagation of lipid peroxidation chain reactions; and (2) through the antioxidant activity of BEO in the coating, which scavenge reactive oxygen species (ROS) and protect membrane lipids from peroxidative degradation. Thus, combined with Figure 5c, the CMTG + PE coating inhibited the respiration, reduced the production of MDA, maintained membrane structural integrity, delayed the softening and senescence, and maintained the quality of the cherry tomatoes.

## 4. Conclusions

In this study, the structure of sodium caseinate was modified and characterized using tannic acid. The results demonstrated that the ability of sodium caseinate to stabilize Pickering emulsions was enhanced following TA modification. Subsequently, a Pickering emulsion was prepared using SC-TA nanocomposites as solid particles and BEO as the oil phase, and a CMTG + PE coating solution was formulated using CMTG as the coating substrate. The structural properties (SEM, FTIR), thermal stability (TGA), WVP, mechanical properties (TS and EAB), antimicrobial activity, and antioxidant activity of the films were investigated. The results indicated that the incorporation of PE into the composite films yielded significant multifunctional improvements, as evidenced by a marked reduction in WVP coupled with enhanced mechanical strength, antimicrobial performance, and antioxidant activity. The CMTG + PE coating was applied to the preservation of cherry tomatoes, where it effectively inhibited the growth of *Alternaria alternata*, delayed changes in weight loss, firmness, respiration rate, TSS, TA, VC, lycopene and MDA content, and extended the shelf life of the fruit. Given that the CMTG + PE coating functions through physical barrier effects and antimicrobial activity, its preservation mechanism is not limited to a specific fruit type, suggesting potential applicability to other fruits. However, it should be noted that this study was conducted under controlled laboratory conditions and did not include actual supply chain simulations (such as temperature fluctuations, transportation and handling). Further research is necessary to evaluate the coating’s performance under practical storage conditions, assess consumer acceptability, and verify its efficacy on a wider range of produce. In conclusion, the Pickering emulsion coating based on carboxymethyl tara gum and containing bergamot essential oil represents a promising candidate for bioactive packaging material, though additional validation is required to assess its commercial feasibility.

## Figures and Tables

**Table 1:** Secondary structure composition of solid particles: α-helix, β-sheet, β-turn, and random coil content.

Nanoparticles	α-Helix (%)	β-Sheet (%)	β-Turn (%)	Random Coil (%)
SC	35.93	0.70	31.62	31.75
SC-TA	38.96	2.60	28.72	29.72

**Table 2:** Particle size distribution, PDI, and zeta potential of solid particles and Pickering emulsions.

Nanoparticles	Size (nm)	PDI	Zeta-Potential (mV)	Pikering Elusions	Size (μm)	PDI	Zeta-Potential (mV)
SC	291.20 ± 3.73 ^a^	0.40 ± 0.07 ^a^	−31.33 ± 0.84 ^b^	SC-PE	2.90 ± 0.28 ^b^	0.54 ± 0.05 ^a^	−35.37 ± 3.17 ^a^
SC-TA	290.83 ± 13.31 ^a^	0.67 ± 0.13 ^b^	−33.03 ± 0.65 ^a^	SC-TA-PE	1.60 ± 0.16 ^a^	0.48 ± 0.01 ^a^	−41.97 ± 0.25 ^b^

Note: Values in the same column with different letters are significantly different (*p* < 0.05).

**Table 3:** Film thickness, WVP, TS, and EAB.

Film Type	Thickness (μm)	WVP (mg/Pa·h·m)	TS (MPa)	EAB (%)
TG	51.00 ± 1.00 ^a^	1.39 ± 0.13 ^c^	4.81 ± 0.81 ^b^	19.22 ± 3.84 ^b^
CMTG	71.00 ± 2.00 ^c^	1.09 ± 0.01 ^b^	11.39 ± 0.29 ^c^	40.89 ± 1.57 ^c^
TG + PE	57.33 ± 0.58 ^b^	1.36 ± 0.13 ^c^	2.59 ± 0.28 ^a^	4.67 ± 0.91 ^a^
CMTG + PE	80.00 ± 1.00 ^d^	0.87 ± 0.02 ^a^	13.27 ± 1.19 ^d^	37.68 ± 3.27 ^c^

Note: Values in the same column with different letters are significantly different (*p* < 0.05).

## Data Availability

The original contributions presented in this study are included in the article. Further inquiries can be directed to the corresponding authors.
